# The Case of the Red Extremities

**DOI:** 10.5811/cpcem.2021.10.52891

**Published:** 2022-01-28

**Authors:** Kelechi Abarikwu, James S. Komara, Andrej Urumov

**Affiliations:** * University of Arizona College of Medicine, Tucson, Arizona; † Mayo Clinic, Department of Emergency Medicine, Phoenix, Arizona

**Keywords:** erythromelalgia, red extremities

## Abstract

**Case Presentation:**

A 37-year-old man with severe obstructive sleep apnea presented to the emergency department with burning pain, redness and swelling in his hands and feet, worsening for several weeks. Pertinent laboratory studies revealed polycythemia.

**Discussion:**

Erythromelalgia is a clinical diagnosis characterized by painful burning, erythema, warmth, and edema usually involving the distal extremities. Therapeutic goals are focused on symptom reduction, while also managing the underlying condition in cases of secondary erythromelalgia. Pharmacological and non-pharmacological therapies have proven to be of limited success.

## CASE PRESENTATION

A 37-year-old man with severe obstructive sleep apnea presented to the emergency department (ED) with burning pain, redness and swelling in his hands and feet (Image), worsening for several weeks. On physical examination, the extremities exhibited a blanching circumferential erythema. The extremities were warm to touch, with a non-pitting edema.

Laboratory evaluation demonstrated a hemoglobin of 19.8 grams per deciliter (g/dL) (reference range: 13.2–16.6 g/dL) and hematocrit of 59.9% (38.3–48.6%), suggestive of polycythemia, presumably secondary to sleep apnea.

## DISCUSSION

Erythromelalgia is a clinical diagnosis characterized by painful burning, erythema, warmth, and edema usually involving the distal extremities. The pain of erythromelalgia may be intermittent, lasting between minutes to days, and is frequently precipitated by heat exposure. Erythromelalgia may occur as a primary or secondary disorder. In its primary form, it has been linked to an autosomal dominant mutation in the sodium voltage-gated channel alpha subunit 9 (SCN9A) gene.^1^ Secondary erythromelalgia occurs as a result of a multitude of conditions, including myeloproliferative disorders, connective tissue diseases, infections, and malignancy.^2^ We postulate that the etiology of erythromelalgia in our patient was secondary to polycythemia.

Therapeutic goals are focused on symptom reduction, while also managing the underlying condition in cases of secondary erythromelalgia. Most therapy has limited efficacy. Non-pharmacological treatments include trigger avoidance, cooling of affected areas, and psychological counseling.^3^ Pharmacological interventions include topical anesthetics, antidepressants, gabapentin, and glucocorticoids. Aspirin has been suggested for treatment in patients with erythromelalgia secondary to myeloproliferative disorders.^4^ Given that our patient’s presenting symptoms were not debilitating, no specific therapy was provided in the ED. Prognosis is dependent on the underlying condition as well as on the patient’s ability to mitigate the symptoms.

**CPC-EM Capsule**

What do we already know about this clinical entity?

*Erythromelalgia is an episodic, painful, pruritic, and edematous erythroderma of the distal part of the extremities with the diagnosis made on clinical grounds*.

What is the major impact of the image(s)?

*The image provides awareness of this condition to the emergency medicine provider*.

How might this improve emergency medicine practice?

*Early recognition of erythromelalgia results in faster initiation of therapy that hastens symptomatic relief and promotes improved patient experience*.

## Figures and Tables

**Image:**
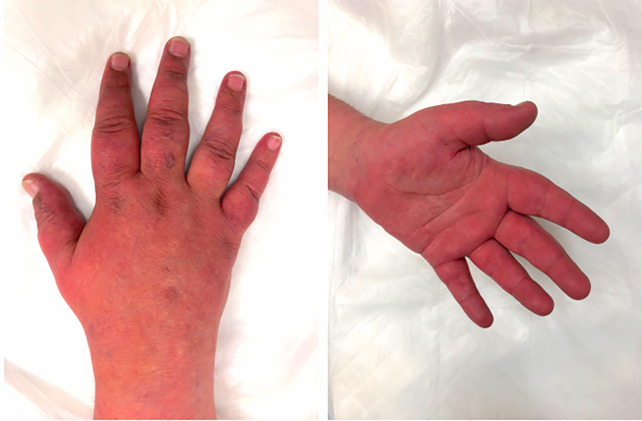
Hands and feet erythema and edema in a patient with erythromelalgia.
